# CHALLENGES IN SELF-MANAGEMENT AMONG OLDER ADULTS WITH HYPERTENSION AND DIABETES

**DOI:** 10.1093/geroni/igz038.1157

**Published:** 2019-11-08

**Authors:** Hyunkyoung Oh, Yura Lee, Wonchan Choi, Zhi Zheng

**Affiliations:** 1 University of Wisconsin-Milwaukee College of Nursing, Milwaukee, Wisconsin, United States; 2 University of Wisconsin-Milwaukee Helen Bader School of Social Welfare, Milwaukee, Wisconsin, United States; 3 University of Wisconsin-Milwaukee School of Information Studies, Milwaukee, Wisconsin, United States; 4 University of Wisconsin-Milwaukee College of Engineering and Applied Science, Milwaukee, Wisconsin, United States

## Abstract

This abstract introduces an ongoing research project that aimed to develop a patient-centered self-management program using health information and technologies for older adults with hypertension and diabetes. The purpose of the project in the first phase was to better understand challenges in self-management faced by older adults with both conditions. A semi-structured and face-to-face interview was conducted to explore the challenges in self-management of the target population living in Milwaukee areas, Wisconsin. Audio recordings were transcribed in verbatim; transcripts were analyzed; and themes were identified. A total of six individuals participated in this study by January 2019. Their age ranged from 56 to 75. Four of them were female; five of them were African Americans; and one was Caucasian. All participants reported more than two additional conditions that were arthritis, cardiovascular diseases, pain, kidney diseases, respiratory diseases, and depression. Most participants were self-managing their conditions mainly by taking prescribed medications. Several themes were emerged as challenges to self-management: monitoring blood pressure and glucose, engagement in physical activity, and healthy eating. Among these, participants reported healthy eating as the most difficult self-managing activity. Majority of participants expressed the need for physical activity support due to pain and/or vision problems known as one of diabetic complications. Understanding challenges and needs of a specific population is the first step for health care providers to support self-management of the patients appropriately. The results of this preliminary study will help health care providers develop effective self-management programs for older adults with both conditions.

